# Pulp Vitality Diagnosis by Means of an Optical Pulp Scanning Device

**DOI:** 10.3390/dj12100326

**Published:** 2024-10-12

**Authors:** Benjamín Briseño Marroquín, Markus Borgschulte, Alexander Savic, Thomas Peter Ertl, Thomas Gerhard Wolf

**Affiliations:** 1 Department of Restorative, Preventive and Pediatric Dentistry, School of Dental Medicine, University of Bern, 3010 Bern, Switzerland; 2 Department of Periodontology and Operative Dentistry, University Medical Center of the Johannes Gutenberg University Mainz, 55131 Mainz, Germany; 3 VDW GmbH, 81737 Munich, Germany; 4 DeguDent GmbH, Dentsply Sirona, 62456 Hanau-Wolfgang, Germany

**Keywords:** flowmetry, oximetry, pulp diagnostic method, pulp vitality

## Abstract

This study aimed to evaluate the diagnostic accuracy of an Optical Pulp Scanning (OPS) device. A total of 421 teeth from 107 emergency patients were investigated. Pulp vitality was assessed using conventional diagnostic methods (ConvDia), cold pulp testing (CPT), and a final diagnosis (FinDia) based on the pulp chamber contents (PCCs) of teeth requiring endodontic treatment (EmeTe). Following ConvDia, OPS screening was performed, endodontic access was prepared, and the FinDia was established. The specificity, sensitivity, as well as positive and negative predictive values of ConvDia, CPT, and FinDia were calculated against the OPS results. The null hypothesis stated that specificity and sensitivity would be equal to or lower than 90% and 80%, respectively. OPS sensitivity was 66.2% and 72.2% (EmeTe) for ConvDia, 66.2% and 69.8% (EmeTe) for CPT, and 76.9% for FinDia. Specificity was 26.4% and 25.5% (EmeTe) for ConvDia, 26.9% and 21.7% (EmeTe) for CPT, and 31.5% for FinDia. These results suggest that, at its current stage of development, the OPS device does not provide an acceptable level of diagnostic accuracy, particularly in vital teeth.

## 1. Introduction

Accurate diagnosis of pulp and adjacent periapical tissues is essential for making informed therapeutic decisions [1]. Currently, this diagnosis relies on evaluating clinical symptoms, conducting oral inspections, reviewing radiographs, and performing pulp ‘vitality’ tests. A correlation between the histopathological status of the pulp and clinical findings, both subjective and objective, can be drawn through a thorough evaluation of the patient’s health history, oral inspection, pulp tests, and radiographic results [2]. However, diagnosing pulp conditions is challenging because conventional pulp vitality tests rely on nerve stimulation to reproduce the symptoms of various pulpal pathologies, which makes the process dependent on the patient’s subjective response [3,4] and the operator’s interpretation [3]. These tests cannot detect pulpal blood flow [3,4], which should be the primary factor in assessing true pulp vitality [5]. Therefore, decisions regarding pulp therapy should be based on objective assessments of blood flow rather than subjective nerve responses [4]. Although conventional cold pulp testing (CPT) is the most commonly used method and is considered reliable [6,7], it cannot be regarded as the ‘ideal’ pulp test. It is invasive and often causes discomfort or pain, which can lead to false positive or false negative results, especially if neighboring oral structures are affected [8]. Measuring pulpal blood circulation and oxygen supply would offer an objective way to differentiate between healthy and unhealthy pulp tissue. Laser Doppler flowmetry (LDF) [4,9,10,11], pulse oximetry (PO), oxygen saturation [12,13], and other non-traditional methods [14] have been investigated to determine for their potential for accurately assessing pulp health status. These light transmission or reflection-based optical methods aim to measure pulpal blood flow and oxygen saturation, providing a quantifiable and non-invasive approach to assessing pulp vitality [4,11]. While vital pulp therapy (VPT) has become increasingly important due to advances in materials and research, there remain significant gaps in knowledge and variations in clinical practice, particularly concerning the need for better diagnostics based on stronger evidence [15] to improve diagnostic outcomes and treatment success. A clinical assessment of the pulp tissue’s cellular structure without opening the tooth is not yet feasible, and despite the advancements of the last century, a reliable method for accurate pulp diagnosis remains unavailable in dentistry [16].

Thus, this study aimed to investigate the diagnostic accuracy of a pulp vitality scanning device (OPS device) compared with clinically conventional and non-conventional pulp diagnostic tests and to generate further clinical data to improve the algorithm’s classification. The null hypothesis postulated that specificity and sensitivity are equal to or smaller than 90% and 80%, respectively.

## 2. Materials and Methods

This single-centered prospective study conducted at the Department of Periodontology and Operative Dentistry, Johannes Gutenberg University, Mainz, Germany, is consistent with the EN-ISO standard —Clinical investigation of medical devices for human subjects—Good Clinical Practice in the form at the time of submission to the ethics committee (https://www.iso.org/standard/71690.html; last access 9 October 2024). It was approved by the independent ethics committee of the State Chamber of Physicians of Rhineland-Palatinate and German Federal Institute of Drugs and Medical Devices, Germany. All procedures were carried out in accordance with the relevant guidelines and regulations. Five certified and calibrated operators (Interdisciplinary Center for Clinical Trials, Mainz, Germany) were responsible for the clinical procedures. Operator calibration consisted of assessment and training regarding clinical conventional diagnostic parameters using the Optical Pulp Scanning (OPS/VDW GmbH, Munich, Germany) device, which was developed specifically for this study. The ethics committee also stipulated that the OPS-derived pulp status results should not influence the clinical treatment decisions. The pulp chambers were clinically inspected visually using 2.5-times magnification.

A total of 421 teeth from 107 emergency dental patients were included in this study. Inclusion criteria were teeth conventionally diagnosed (ConvDia) as having acute or chronic pulpitis, non-vital pulp, or periapical tissue showing a radiological pathological condition, or teeth that were symptomatically endodontically treated (EndTe). These teeth were classified as ‘emergency teeth’ (EmeTe). Additionally, mesially and distally located teeth were included as control teeth when feasible. An access cavity was prepared only for EmeTe requiring endodontic treatment based on ConvDia. Symptomatically treated EndTe were excluded from the EmeTe group. The study aimed to include at least 200 teeth diagnosed as non-vital through ConvDia, with a minimum of 100 being non-EndTe. Exclusion criteria were individuals who were not able to give informed consent, pregnant and nursing patients, those with primary dentition, those with a missing endodontic treatment indication, individuals unable to comply with the study protocol due to a medical, social, or psychological condition, and individuals who were financially or otherwise dependent on the research or sponsoring institution. The distinction between acute and chronic pulpitis was identified by means of pain symptoms. If there was pain, it was classified as acute pulpitis, and no pain was classified chronic pulpitis (often an incidental finding). In cases of acute pulpitis, the tooth could be either vital or non-vital, whereas chronic pulpitis always indicated a non-vital tooth.

The pulp health status was diagnosed using conventional pulp diagnostic (ConvDia) tests, cold pulp testing (CPT), and a “final” diagnosis (FinDia) according to the in-situ pulp chamber content (PCC) observations after endodontic access (Table 1). CPT (−44 °C; Provotest; Hoechst-Pharma AG, Zurich, Switzerland) was performed with a foam pellet applied to the vestibular or palatal surface of the tooth. Uncertain CPT responses were repeated after 30 s. The FinDia findings either confirmed or revoked the ConvDia and were correspondingly electronically recorded. The electrical test (Vitality Scanner, Sybron Endo, Kerr Corporation, Gilbert, AZ, USA) was used when the cold test produced unclear results. Percussion testing, gingival status, and diagnostic radiographs were re-evaluated by an unbiased observer only when ConvDia or FinDia results did not align with those obtained from the Optical Pulp Scanning (OPS) device. EmeTe, EndTe, and control teeth were screened with the OPS device following CPT and ConvDia, and the results were recorded in a double-blinded manner (patient codes anonymized).

**Figure 1:**
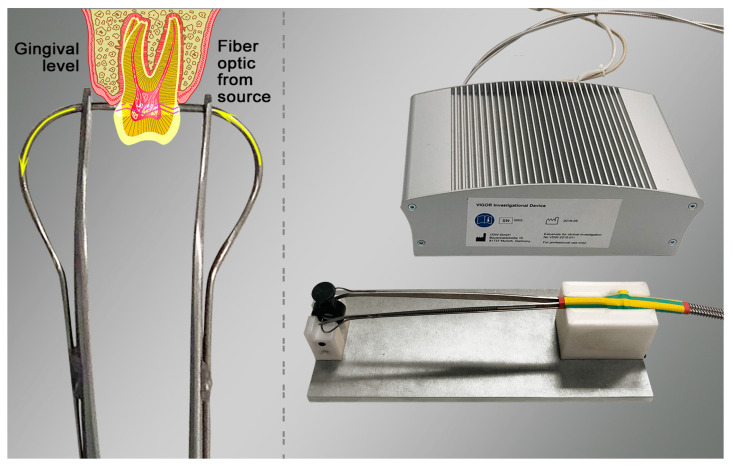
A light source and computer terminal for the Optical Pulp Scanning (OPS) device (**top right**) were developed for this investigation. Smooth and fine probe tips (Ø 0.2 mm; detail, **left**) were fixed to dental forceps, thus allowing a stable placement in the tooth to be measured (**bottom right**). The diagram detail (**left**) depicts the placement of the glass fiber probes of the Optical Pulp Scanning (OPS) device at the gingiva level (G0), and the light dispersion going into the tooth pulp and being conducted to the detector using optical fibers.

The OPS device’s working principle is based on transmitted light spectroscopy in a wavelength between 450 nm and 750 nm and a spectral response analysis. The OPS handpiece is provided by light-conductive fibers (Ø 0.2 mm) from an LED source to a spectrometer (Figure 1). The probes were optically shielded with stainless-steel tubes and transparent single-use plastic sleeves to avoid cross-contamination. Through a specific embedded software, developed for this purpose, the OPS automatically adjusted the measuring time, depending on the tooth width and translucency. The spectral data were obtained within 10 to 6000 ms and recorded in an electrically unplugged notebook using a USB connection (Figure 1).

A specific algorithm was developed to analyze the measured light transmission spectra in a wavelength range between 500 and 650 nm, thus allowing for the determination of the maximum absorption of hemoglobin in its oxygenated (λ = 541 nm and λ = 577 nm) and deoxygenated (λ = 555 nm) states, and other spectral features associated with the pulp status. Spectral features were extracted by differentiation (first and second derivative) and integration in determined wavelength areas. Four features were extracted from the respective spectral curve integration and five features were from the first and second derivatives. From those nine spectral features, eight combinations of two features each were combined in x–y diagrams (one feature on the x- and one on the y-axis, respectively). From a subset of the patient measurements with a known diagnosis, a range between the min and max values on both diagram axes was defined, which was typical for a vital pulp or a non-vital pulp. These value ranges on the y and x axes together defined rectangle “boxes” in the x–y diagram, with one box containing the most probable values for vital pulp and the other box for non-vital pulp, yet without an overlap of both boxes. Other areas in the diagrams were considered “undefined”. This was carried out for all eight combinations selected from the nine spectral features. If the data points were located in all eight diagrams within the vital box, then the probability for this measurement to be vital was considered as 1. If the data points were found within only, e.g., four vital boxes, the probability of being vital was considered as 0.5. Through comparison of the vital and non-vital probabilities, a decision was taken to determine it as either a vital, non-vital, or unclear pulp diagnosis. An unclear pulp diagnosis was defined when the probability of being vital and non-vital was approximately the same or when most data points were located outside the predefined rectangular areas.

The embedded software started a measurement by itself and recorded a successful measurement advised with a signal tone when the specific parameters (signal stability and intensity) were within the predefined range. During the OPS measurement, the operator’s voice including the anonymized patient record, tooth number being examined, and probe site placement was also recorded. The OPS conductive fiberglass probes were placed in the buccal and lingual aspects of the teeth at the gingival margin (G0), under a crown margin where this was existing (Figure 1), to be able to avoid light conduction interferences during the measurements. A rather short training period was required to understand and master how the probes should effectively be placed for the anterior and posterior teeth. After completing the OPS measurements, a patient’s procedure comfort/discomfort was recorded according to the VAS scale. The spectral intensity signal was split into spectral regions and analyzed (Excel 16.40 Microsoft, Redmond, WA, USA) as follows:

- by integrating the intensity within interesting spectral regions (e.g., peak oxygenated and non-oxygenated blood regions) and
- by calculating the spectral absorption curves slope and turning points using differentiation within the various spectral regions.

The different integration and differentiation results were then fed into a classifier with predefined boundaries. The pulp/tooth status decision (vital, non-vital, or an EndTe) was derived from weighted values, which were calculated from the raw results inside or outside of areas separated by boundaries.

An access cavity was prepared after OPS measurement completion, and the FinDia was established after removing the pulp chamber roof of the EmeTe and its contents were visually inspected (Table 1). EndTe underwent ConvDia and OPS measurement and they were protocoled correspondingly, being classified as negative with the CPT. The results obtained with the OPS device were defined as being of a vital, non-vital, not-EndTe, non-vital EndTe, or unknown (pulp health) status. The specificity, sensitivity, and positive and negative predictive values of the OPS device were calculated according to the results obtained with the CPT, ConvDia, and FinDia, which were defined as reference (gold) standards. Three research groups were created for purposes of comparison: ConvDia/OPS (all teeth and EmeTe), FinDia/OPS (EmeTe), and CPT/OPS (all teeth and EmeTe). For a possible generalization of the results, a post-hoc sample size calculation was carried out (www.openepi.com; last access 7 October 2024) to confirm whether the analyzed sample was statistically significant [17]. The null hypothesis postulated that specificity and sensitivity are equal to or smaller than 90% and 80%, respectively.

## 3. Results

**Figure 2:**
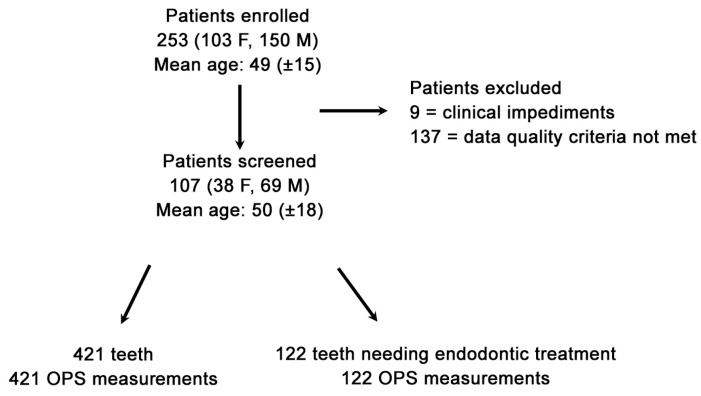
Flow diagram of collected data of all teeth when considering the clinical and Optical Pulp Scanning (OPS) device exclusion parameters at the gingival measuring level (G0).

Due to the research’s stringent stipulated inclusion conditions, 146 out of 253 patients seeking dental emergency treatment were excluded, attributable to clinical impediments or Optical Pulp Scanning (OPS) device deficiencies (Figure 1). The final validated datasets were 107 patients (Power = Φ(13.556) = 1 = 100% power analyses) with 543 OPS measurements made at the gingival line (G0). The specifically developed algorithm allowed the identification of lower-grade quality data that was impacted by any interference source (e.g., neighboring fluorescent light source fingerprints); thus, out of the validated datasets, 107 patients with a total of 421 OPS measurements (122 emergency teeth and 299 control teeth) who met the stringent inclusion criterion (Figure 2) were analyzed. A total of 139 measurements were not included in the final analysis due to an OPS measurement error or device feedback. A complete patient OPS screening took, at most, five minutes and was performed immediately after the patient had been recruited and ConvDia had been carried out. As expected, after this point, no further dropouts occurred.

Due to the unexpected OPS low specificities and sensitivities of EndTe, and although the results were identifiable as such, they were classified and statistically analyzed as negative teeth in the corresponding group. The sensitivities, specificities, and positive and negative predictive OPS values were calculated assuming that the CPT, ConvDia, or FinDia were 100% correct. A summary of the results of all teeth divided into tooth-type groups according to the compared gold standard is given in Table 2, Table 3, Table 4, Table 5, Table 6 and Table 7. According to the VAS scale (0–10), 92 (86.0%) patients reported no discomfort (scale value = 0) and 6 (5.6%), 8 (7.5%), and 1 (0.9%) patients reported a relatively low discomfort level of 1, 2, and 3, respectively, according to the VAS scale, during the OPS procedure (n = 107). The mean VAS-scale response was 0.23%. The OPS diagnostic/screening procedure proved to be uncomplicated and rapid, and measurements under crown margins were feasible and effortless.

## 4. Discussion

### 4.1. Dental Pulp Vitality Diagnosis

Dental pulp vitality diagnosis relies on systematic information gathering through questioning, clinical examination, and logical evaluation [5], which is essential for therapy decisions. However, the lack of modern pulp diagnostic technology is a limitation of the dental profession [14,15,16]. This study aimed to develop an Optical Pulp Scanning (OPS) device for objective, effortless, and non-invasive pulp health assessment. Following a successful pretrial with a small group size (n = 12), the main trial was conducted using recommended diagnostic accuracy designs, participant random allocation, and concealment [18]. Electric, cold pulp (CPT), and heat testing [4,13,19,20], along with endodontically treated teeth (EndTe) [11,13] and in situ pulp chamber content (PCC) observations (FinDia) [4], served as reference standards to assess the accuracy of the OPS device [14].

### 4.2. Comparison with Conventional Diagnostic Methods

Although CPT is accurate, it cannot be used as a standalone diagnostic method [21]. Therefore, it was included as part of a composite reference standard alongside ConvDia and FinDia. A control tooth (neighboring tooth) was used to verify questionable responses. Control and EndTe were compared with the OPS results (Table 1). Unlike previous studies [8,22], this study used a composite standard of routine and non-routine diagnostic methods to define the “true/actual” pulp health status (CPT, ConvDia, and FinDia) [23]. Imperfect reference standards can bias accuracy estimates [24], so FinDia was not considered a definitive diagnostic method [11]. The study’s parameters (Table 1), agreed upon by five endodontic clinicians, should be interpreted cautiously as subjective support for the OPS results. Laser Doppler flowmetry (LDP) [25] and pulse oximetry (PO) [26] face technical issues such as non-pulpal “noise” [13], probe size/shape [19], signal detection limits [27], and motion artifacts [28]. As a result of these challenges, 139 OPS measurements were excluded, primarily because of background light interference or operator error, while only nine were excluded due to record discrepancies. Despite these exclusions, the remaining data allowed for reliable statistical analysis. The OPS pre-defined algorithm only allowed measurements to be recorded when specific thresholds were met, reducing false positive and false negative results. As the dataset was restricted, especially with few non-vital/non-endodontically treated teeth), the data obtained will facilitate future improvements to the algorithm, enhancing reliability.

### 4.3. Population Age and Pulp Response Discrepancies

The age range investigated in this study (18 to 90 years; mean 50 ± 18) was similar to ranges used in previous LDF [25,29] and pulse oximetry (PO) [4,19] studies. However, unlike earlier findings [30], the OPS device results showed no false responses in elderly patients. This discrepancy may be attributable to the sample size or the types of teeth examined. While thinner enamel and dentin improve signal-to-noise ratios for pulpal blood flow measurements [31], the model used here does not mimic a typical clinical situation where most teeth have intact enamel and dentin. Furthermore, enamel scatters and blurs the incoming light direction; yet, when the light source reaches the dentinal tubules, it is still dominant, and major portions of light are gathered towards the pulp [32]. However, some light may scatter through the surrounding gingival or periodontal tissue, thus including a signal information from vascularized tissue competing with the blood flow signal from the pulp. Future OPS device improvements should focus on differentiating signals from the pulp and surrounding tissues, as well as addressing light scattering behavior in enamel and dentin.

### 4.4. Recognizing Endodontically Treated Teeth and Limitations of Diagnostic Methods

The OPS device was originally designed to recognize endodontically treated teeth (EndTe), but sensitivity was unexpectedly low. Since this was not the primary goal of the OPS device at this stage, EndTe was classified as non-vital, and detailed results will be reported after technological improvements. The clinical assessment of pulp status via naked-eye inspection was unquantifiable, therefore offering limited clinical relevance. Similarly, CPT, though established, remains a subjective method. In this study, pulp content was treated as a complementary, not primary, diagnostic tool. The electrical test was excluded due to the presence of too many crowns, despite its good repeatability [29,30,33]. FinDia, though used in this study, has significant limitations as a diagnostic reference due to its invasive nature and subjective interpretation. Future research should focus on non-invasive methods to improve diagnostic accuracy and reliability in assessing pulp vitality.

### 4.5. Measurement Stability and Light Penetration

Similar to LDF and PO, reliable OPS measurements require stable optical fiber positioning and controlled movement, which were addressed by the OPS software. Only 0.6% of excluded teeth were due to operator errors such as learning curve deficiencies. Measurement time, varying from 10 to 6000 ms (mean 432 ms), was not deemed significant. Another limitation of LDF and PO is that restored teeth can distort light penetration or reflection [25], but this was resolved by the OPS probe design, allowing stable measurements at the gingival level (G0) under cervical restorations (Figure 1). Measurements at the enamel level were not included in this study because results were inferior to G0, and measuring at the enamel level was often not feasible with crowns or resin restorations. Initial concerns regarding potential pulp calcifications suggested G0 measurements could be challenging, but the study demonstrated their consistent feasibility. Measurements 2 mm above the cervical margin (G2) were also investigated but excluded, as no statistical differences were found between G0 and G2.

### 4.6. Future Developments and Optimizing OPS Sensitivity and Specificity

The periodontal pocket depth was not relevant in this investigation, but inflamed gingiva was considered, as it could increase tissue perfusion signals not originating from the pulp, potentially causing false readings. The potential for measurements at G2, due to a reduced gingival and periodontal tissue blood flow influence, to yield higher sensitivity and specificity could have led to the misclassification of vital teeth. However, no such differences were observed; this is likely to be because the algorithm was optimized for G0 measurements. Previous studies reported higher sensitivities and specificities with laser Doppler flowmetry (LDF) [11,25] and pulse oximetry (PO) [4,11,19], but an LDF review highlighted biases and design flaws [10]. In this study, OPS results showed lower sensitivities (77.0–66.2%) and specificities (31.3–21.7%) than expected, likely due to strict adherence to randomized diagnostic accuracy requirements. Predictive values, influenced by a disease prevalence of 39% for CPT and electrical pulp tests [8], and 52% for PCC observations [4], varied between this study and others (ConvDia = 58.0%, CPT = 57.5%, FinDia = 53.8%), making direct comparison difficult. One advantage of PO, LDF, and OPS over thermal or electric tests is their objectivity, as they do not rely on invasive stimuli, reducing sampling errors [6]. This is supported by the non-noxious nature of OPS, which resulted in low patient discomfort (VAS mean = 0.23%) [34]. Additionally, these non-invasive methods allow quick, complete pulp status screening.

This study did not account for factors such as large restorations or patient age, which may reduce gingival blood flow interference and influence the diagnosis. These parameters should be considered in future research to enhance sensitivity and specificity since possible volume reductions could positively influence the diagnostic field, eliminating the gingival blood flow influence. This study focused on spectrally resolved light transmission, which marks the beginning of OPS development for pulp vitality diagnosis. Data from this study, based on strict inclusion criteria, will support future OPS device improvements at the G0 and G2 levels, enhancing diagnostic accuracy. Further OPS device developments, including reducing gingival tissue signal interference, refining algorithms, and adjusting the wavelength, are expected to improve the sensitivity and specificity.

## 5. Conclusions

The Optical Pulp Scanning (OPS) device demonstrated sensitivities and specificities lower than those expected under the null hypothesis and reference standards. Within the limitations of this study, the results suggest that, at its current stage of development, the OPS device is not capable of providing a clinically acceptable level of diagnostic confidence, particularly for non-healthy pulps, which were inaccurately classified as vital due to the current OPS classifier boundary settings.

## Figures and Tables

**Table 1:** Research parameters foreseen, recorded for all teeth investigated and of the pulp chamber content (FinDia) observations (VAS = visual analog scale; 0 = none, 10 = maximum; mobility grade I corresponds to straight perceptible, grade II to a visible mobility, and grade III to mobility on lip and tongue pressure or mobility in the vertical plane; ++ = extreme reaction; EndTe = endodontically treated tooth; + = positive; − = negative; N/A = Not applicable).

Clinical parameters investigated (ConvDia)	
Inclusion date:	Electric test: −, +, ++.
Patient ID:	Percussion test: −, +, ++.
Teeth included: emergency tooth and two controls.	Test cavity: −, +, ++.
Sex:	Gingiva status: N/A, inflamed, swollen, retracted.
Age:	Periodontal status: N/A, deep pockets.
Pain intensity: 1 to 10 VAS scale.	Mobility degree: 0, I, II, III.
Filling status: N/A, filling, crown, fracture, caries.	Radiographic findings: N/A, apical lesion, lateral lesion, caries, fracture, root canal treatment.
Filling extension: 1 to 5 surfaces.	Radiographic caries findings: 1 to 5 surfaces.
Filling localization: crown, mesial, distal, occlusal, buccal.	Clinical conventional diagnosis: healthy, non-vital, acute pulpitis, chronic pulpitis, necrosis.
Filling material: amalgam, composite, glass ionomer, metal-ceramic, ceramic, provisional material.	Access cavity findings: N/A, healthy, inflamed, necrotic, purulent.
Sensibility to cold: −, +, ++.	Optical device diagnosis: vital, non-vital, non-endodontically treated, non-vital-EndTe, unknown (pulp health) status.
Final diagnosis (FinDia) criteria of the pulp chamber contents (PCCs)	
N/A: non-pathological bleeding observed after endodontic access.	Necrotic: empty pulp chamber, darkened or unidentifiable pulp tissue remnants.
Inflammation: an increased blood flow was observed immediately after opening the pulp chamber.	Purulent: purulent blood or pus was observed after opening the pulp chamber or after slight root canal probing.

**Table 2:** Summary of sensitivity, specificity, and positive (PPV) and negative (NPV) predictive values of the pulp conventional diagnosis (ConvDia) results and Optical Pulp Scanning (OPS) device measurements (absolute values) of the different investigated groups obtained from 107 individuals at the gingival measuring level (G0). Teeth with an endodontic treatment (EndTe) were considered non-vital teeth (PIT = positive index test; NIT = negative index test; + cond. = positive condition; − cond. = negative condition; TP = true positive; TN = true negative; FP = false positive; FN = false negative).

ConvDia		OPS				+ Cond.		− Cond.
Vital	334	Vital	221	PIT	TP (Vi +; ConvDia +)	221	FP (Vi +, ConvDia −)	64
		Non-vital	47	NIT	FN (Vi −, ConvDia +)	113	TN (Vi -, ConvDia −)	23
		EndTe	66	Sensitivity	66.17%			
Non-vital	52	Non-vital	8	PPV	77.54%			
		Vital	38	Specificity	26.44%			
		EndTe	6	NPV	16.91%			
EndTe	35	Non-vital	4	Prevalence	79.33%			
		Vital	26					
		EndTe	5					
Total teeth measurements included in the investigation at the G0 level (n = 421).								
Vital	54	Vital	39	PIT	TP (Vi +; ConvDia +)	39	FP (Vi +, ConvDia −)	52
		Non-vital	5	NIT	FN (Vi −, ConvDia +)	15	TN (Vi −, ConvDia −)	16
		EndTe	10	Sensitivity	72.22%			
Non-vital	52	Non-vital	8	PPV	42.86%			
		Vital	38	Specificity	25.53%			
		EndTe	6	NPV	51.61%			
EndTe	16	Non-vital	1	Prevalence	44.26%			
		Vital	14					
		EndTe	1					
Total emergency teeth measurements at the G0 level (n = 122).								

**Table 3:** Summary of the sensitivity, specificity, and positive (PPV) and negative (NPV) predictive values and prevalence of the pulp conventional diagnosis (ConvDia) results and Optical Pulp Scanning (OPS) device measurements (absolute values) of anterior, premolar, and molar teeth obtained from 107 individuals at the gingival measuring level (G0). Teeth with an endodontic treatment (EndTe) were considered non-vital teeth.

ConvDia		OPS		ConvDia		OPS		ConvDia		OPS	
Anterior teeth (13–43; n = 100)				Premolars (14–44; n = 152)				Molars (16–48; n = 169)			
Vital	79	Vital	55	Vital	125	Vital	61	Vital	130	Vital	105
		Non-vital	12			Non-vital	27			Non-vital	8
		EndTe	12			EndTe	37			EndTe	17
Non-vital	17	Non-vital	1	Non-vital	11	Non-vital	3	Non-vital	24	Non-vital	4
		Vital	4			Vital	7			Vital	17
		EndTe	12			EndTe	1			EndTe	3
EndTe	4	Non-vital	0	EndTe	16	Non-vital	2	EndTe	15	Non-vital	2
		Vital	4			Vital	11			Vital	11
		EndTe	0			EndTe	13			EndTe	2
Sensitivity		69.62%		Sensitivity		48.80%		Sensitivity		80.77%	
PPV		75.34%		PPV		77.22%		PPV		78.95%	
Specificity		14.29%		Specificity		33.33%		Specificity		28.21%	
NPV		11.11%		NPV		12.33%		NPV		30.56%	
Prevalence		79.00%		Prevalence		82.24%		Prevalence		76.92%	
Total teeth measurements (divided into tooth types) included in the investigation at the G0 level.											
Anterior teeth (13–43; n = 29)				Premolars (14–44; n = 28)				Molars (16–48; n = 65)			
Vital	11	Vital	7	Vital	11	Vital	9	Vital	32	Vital	23
		Non-vital	1			Non-vital	0			Non-vital	4
		EndTe	3			EndTe	2			EndTe	5
Non-vital	17	Non-vital	1	Non-vital	11	Non-vital	3	Non-vital	24	Non-vital	4
		Vital	14			Vital	7			Vital	17
		EndTe	2			EndTe	1			EndTe	3
EndTe	1	Non-vital	0	EndTe	6	Non-vital	0	EndTe	9	Non-vital	1
		Vital	1			Vital	6			Vital	7
		EndTe	0			EndTe	0			EndTe	1
Sensitivity		63.64%		Sensitivity		81.82%		Sensitivity		71.88%	
PPV		31.82%		PPV		40.91%		PPV		48.94%	
Specificity		16.67%		Specificity		23.53 %		Specificity		27.27%	
NPV		42.86%		NPV		66.67%		NPV		50.00%	
Prevalence		37.93%		Prevalence		32.39%		Prevalence		49.23%	
Total emergency teeth measurements (divided into tooth types) at the G0 level.											

**Table 4:** Summary of the sensitivity, specificity, and positive (PPV) and negative (NPV) predictive values of the pulp cold test (CPT) results and Optical Pulp Scanning (OPS) device measurements (absolute values) of the different investigated groups obtained from 107 individuals at the gingival measuring level (G0). Teeth with an endodontic treatment (EndTe) were considered non-vital teeth (PIT = positive index test; NIT = negative index test; + cond. = positive condition; − cond. = negative condition; TP = true positive; TN = true negative; FP = false positive; FN = false negative).

CPT		OPS				+ Cond.		− Cond.
Vital	328	Vital	217	PIT	TP (Vi +; ConvDia +)	217	FP (Vi +, ConvDia −)	68
		Non-vital	46	NIT	FN (Vi −, ConvDia +)	111	TN (Vi −, ConvDia −)	25
		EndTe	65	Sensitivity	66.16%			
Non-vital	93	Non-vital	13	PPV	76.14%			
		Vital	68	Specificity	26.88%			
		EndTe	12	NPV	18.38%			
				Prevalence	77.91%			
Total teeth measurements included in the investigation at the G0 level (n = 421).								
Vital	53	Vital	37	PIT	TP (Vi +; ConvDia +)	37	FP (Vi +, ConvDia −)	54
		Non-vital	6	NIT	FN (Vi −, ConvDia +)	16	TN (Vi −, ConvDia −)	15
		EndTe	10	Sensitivity	69.81%			
Non-vital	69	Non-vital	8	PPV	40.66%			
		Vital	54	Specificity	21.74%			
		EndTe	7	NPV	48.39%			
				Prevalence	43.44%			
Total emergency teeth measurements at the G0 level (n = 122).								

**Table 5:** Summary of the sensitivity, positive (PPV), and negative (NPV) predictive values and prevalence of the cold pulp test (CPT) results and Optical Pulp Scanning (OPS) device measurements (absolute values) of anterior, premolar and molar teeth obtained from 107 individuals at the gingival measuring level (G0). Teeth with an endodontic treatment (EndTe) were considered non-vital teeth.

CPT		OPS		CPT		OPS		CPT		OPS	
Anterior teeth (13–43; n = 100)				Premolars (14–44; n = 152)				Molars (16–48; n = 169)			
Vital	78	Vital	55	Vital	122	Vital	58	Vital	128	Vital	104
		Non-vital	11			Non-vital	27			Non-vital	8
		EndTe	12			EndTe	37			EndTe	16
Non-vital	22	Non-vital	2	Non-vital	30	Non-vital	5	Non-vital	41	Non-vital	6
		Vital	18			Vital	21			Vital	29
		EndTe	2			EndTe	4			EndTe	6
Sensitivity		70.51%		Sensitivity		47.54%		Sensitivity		81.25%	
PPV		75.34%		PPV		73.43%		PPV		78.20%	
Specificity		18.18%		Specificity		30.00%		Specificity		29.27%	
NPV		14.81%		NPV		12.33%		NPV		33.33%	
Prevalence		78.00%		Prevalence		80.26%		Prevalence		75.74%	
Total teeth measurements (divided into tooth types) included in the investigation at the G0 level.											
Anterior teeth (13–43; n = 29)				Premolars (14–44; n = 28)				Molars (16–48; n = 65)			
Vital	13	Vital	9	Vital	10	Vital	7	Vital	30	Vital	21
		Non-vital	1			Non-vital	1			Non-vital	4
		EndTe	3			EndTe	2			EndTe	5
Non-vital	16	Non-vital	1	Non-vital	18	Non-vital	2	Non-vital	35	Non-vital	5
		Vital	13			Vital	15			Vital	26
		EndTe	2			EndTe	1			EndTe	4
Sensitivity		69.23%		Sensitivity		70.00%		Sensitivity		70.00%	
PPV		40.91%		PPV		31.82%		PPV		44.68%	
Specificity		18.75%		Specificity		16.67%		Specificity		25.71%	
NPV		42.86%		NPV		50.00%		NPV		50.00%	
Prevalence		44.83%		Prevalence		35.71%		Prevalence		46.15%	
Total emergency teeth measurements (divided into tooth types) at the G0 level.											

**Table 6:** Summary of the sensitivity, specificity, and positive (PPV) and negative (NPV) predictive values of the final diagnosis (FinDia) results and Optical Pulp Scanning (OPS) device measurements (absolute values) of the different investigated groups obtained from 107 individuals at the gingival measuring level (G0). Teeth with an endodontic treatment (EndTe) were considered non-vital teeth (PIT = positive index test; NIT = negative index test; + cond. = positive condition; − cond. = negative condition; TP = true positive; TN = true negative; FP = false positive; FN = false negative).

FinDia		OPS				+ Cond.		− Cond.
Vital	52	Vital	40	PIT	TP (Vi +; ConvDia +)	40	FP (Vi +, ConvDia −)	37
		Non-vital	6	NIT	FN (Vi −, ConvDia +)	12	TN (Vi −, ConvDia −)	17
		EndTe	6	Sensitivity	76.92%			
Non-vital	54	Non-vital	7	PPV	51.95%			
		Vital	37	Specificity	31.48%			
		EndTe	10	NPV	58.62%			
				Prevalence	49.06%			
Total emergency teeth measurements at the G0 level in which an access cavity was prepared (n = 107).								

**Table 7:** Summary of the sensitivity, specificity, and positive (PPV) and negative (NPV) predictive values and prevalence of the pulp cavity contents (FinDia) results and Optical Pulp Scanning (OPS) device measurements (absolute values) of anterior, premolar and molar teeth obtained from 107 individuals at the gingival measuring level (G0).

FinDia		OPS		FinDia		OPS		FinDia		OPS	
Anterior teeth (13–43; n = 29)				Premolars (14–44; n = 22)				Molars (16–48; n = 56)			
Vital	9	Vital	8	Vital	10	Vital	9	Vital	33	Vital	23
		Non-vital	0			Non-vital	0			Non-vital	6
		EndTe	1			EndTe	1			EndTe	4
Non-vital	20	Non-vital	13	Non-vital	12	Non-vital	3	Non-vital	23	Non-vital	2
		Vital	3			Vital	7			Vital	17
		EndTe	4			EndTe	2			EndTe	4
Sensitivity		88.89%		Sensitivity		90.00%		Sensitivity		69.70%	
PPV		38.10%		PPV		56.25%		PPV		41.48%	
Specificity		35.00%		Specificity		41.67%		Specificity		15.79%	
NPV		87.50%		NPV		83.33%		NPV		37.50%	
Prevalence		31.03%		Prevalence		45.45%		Prevalence		58.93%	
Total emergency teeth measurements (divided into tooth types) at the G0 level in which an access cavity was prepared.											

## Data Availability

The data contained in this study are available on request from the corresponding author, as they are subject to data protection restrictions.
